# Supplementary material for: Asymmetric framework motion of TCRαβ controls load-dependent peptide discrimination
Source: bioRxiv. 2023 Sep 13:2023.09.10.557064. Preprint. [Version 1] doi: 10.1101/2023.09.10.557064 (PMC10515854; doi:10.1101/2023.09.10.557064)
Supplement: 1 [file NIHPP2023.09.10.557064V1-supplement-1.pdf]

bioRxiv preprint doi: https://doi.org/10.1101/2023.09.10.557064; this version posted September 13, 2023. The copyright holder for this preprint (which was not certified by peer review) is the author/funder, who has granted bioRxiv a license to display the preprint in perpetuity. It is made available under aCC-BY 4.0 International license.

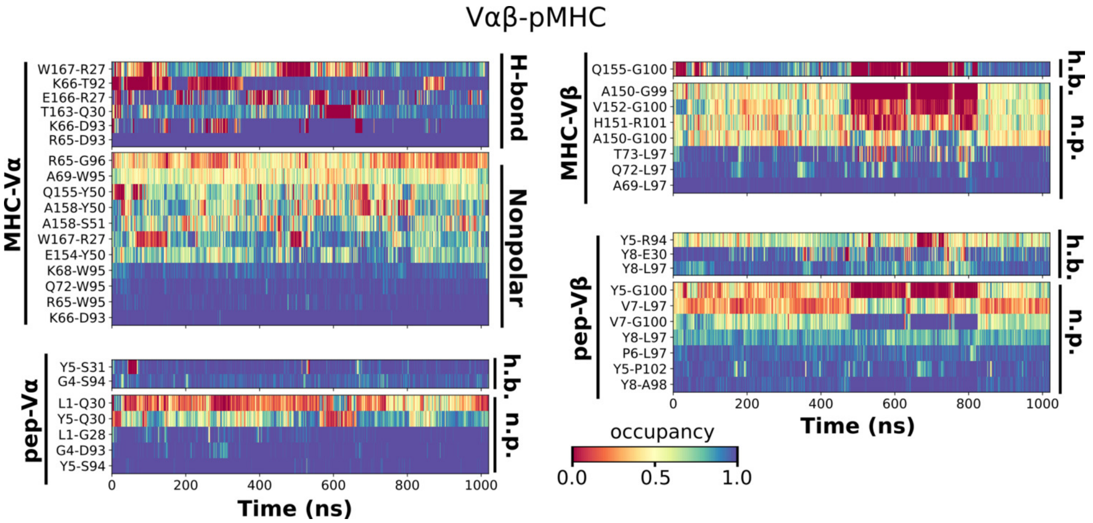

Figure 2—figure supplement 1. Contact occupancy heat maps for V$\alpha\beta$-pMHC. The same occupancy cutoffs as in **Figure 2**C–E were used.

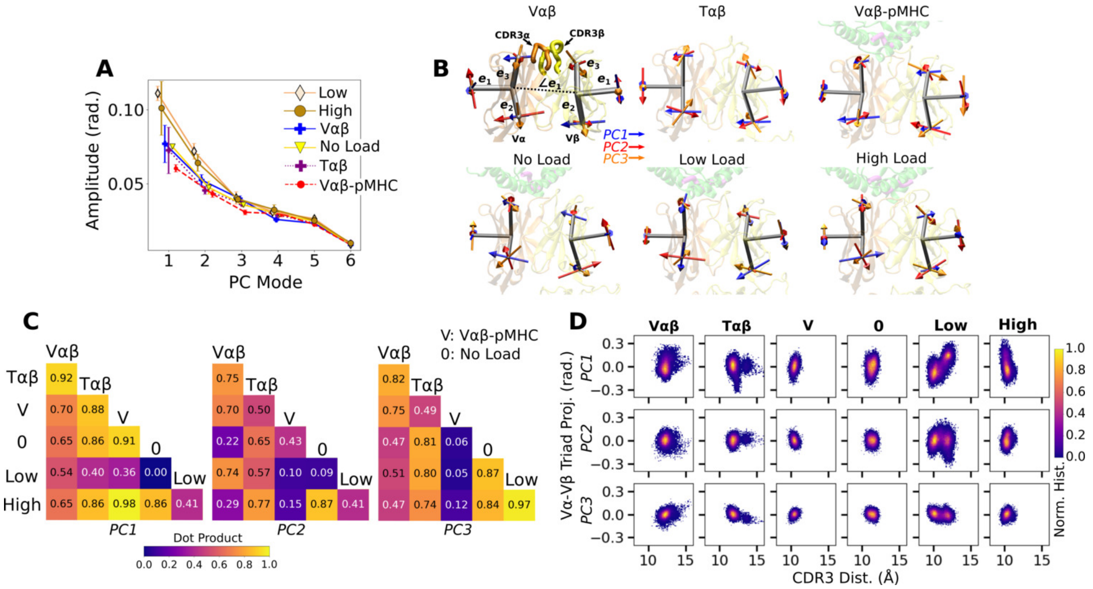

Figure 3—figure supplement 1. PCA of Vα-Vβ motion. (A) PC amplitudes. Bars: std for PCA performed in 3 overlapping intervals from 500 ns to the end of simulation. (B) Direction of motion for the first three PC modes. (C) Absolute values of dot products between the unit PC vectors in listed systems. Values range from 0 (orthogonal) to 1.0 (identical PC directions). (D) 2-dimensional histograms of the projections of the Vα-Vβ triads in each frame onto the first three PC directions versus the CDR3 distance.

bioRxiv preprint doi: https://doi.org/10.1101/2023.09.10.557064; this version posted September 13, 2023. The copyright holder for this preprint (which was not certified by peer review) is the author/funder, who has granted bioRxiv a license to display the preprint in perpetuity. It is made available under aCC-BY 4.0 International license.

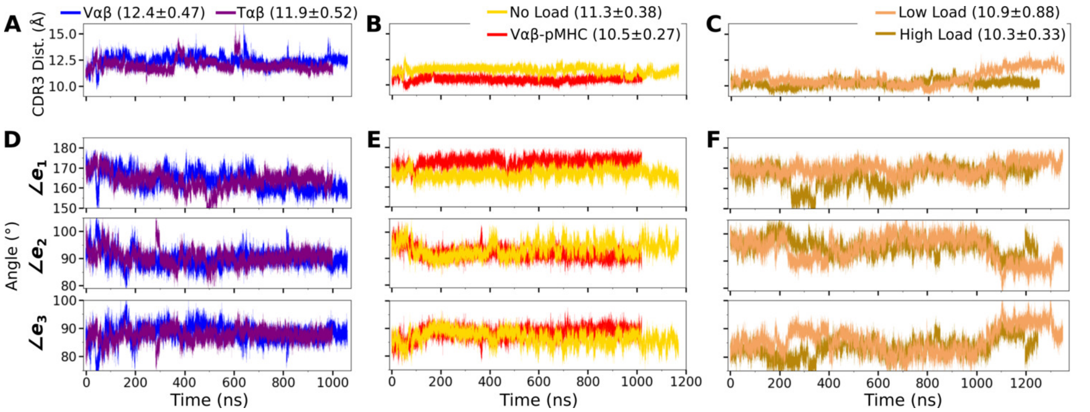

Figure 3—figure supplement 2. Trajectories of the V-module motion. (A–C) CDR3 distances and (D–F) triad angles. (A,D) V$\alpha\beta$ and T$\alpha\beta$, (B,E) V$\alpha\beta$-pMHC and WT^{0}, and (C,F) WT^{low} and WT^{high}. Labels include average and standard deviation of the CDR3 distance after 500 ns.

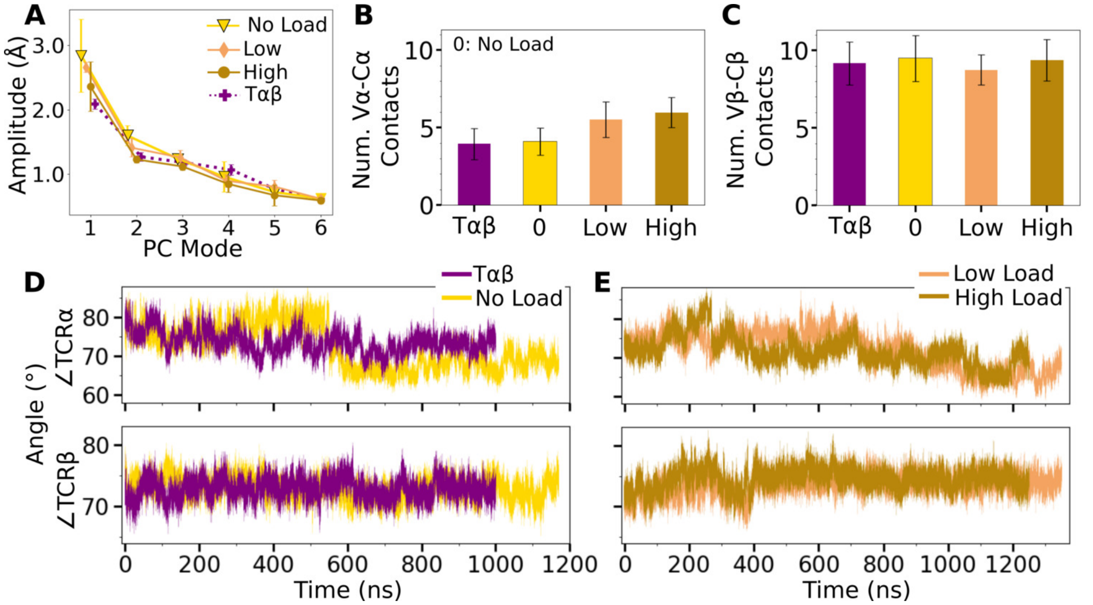

Figure 4—figure supplement 1. V-C PC amplitude and contacts. (A) Amplitude of the first six PCs. Bars: std for PCA performed in 3 overlapping intervals from 500 ns to the end of simulation. (B,C) Number of contacts with greater than 50% average occupancy and 80% maximum instantaneous occupancy for (B) Vα-Cα and (C) Vβ-Cβ. Bars: std. (D,E) Trajectories of hinge angles versus time.

bioRxiv preprint doi: https://doi.org/10.1101/2023.09.10.557064; this version posted September 13, 2023. The copyright holder for this preprint (which was not certified by peer review) is the author/funder, who has granted bioRxiv a license to display the preprint in perpetuity. It is made available under aCC-BY 4.0 International license.

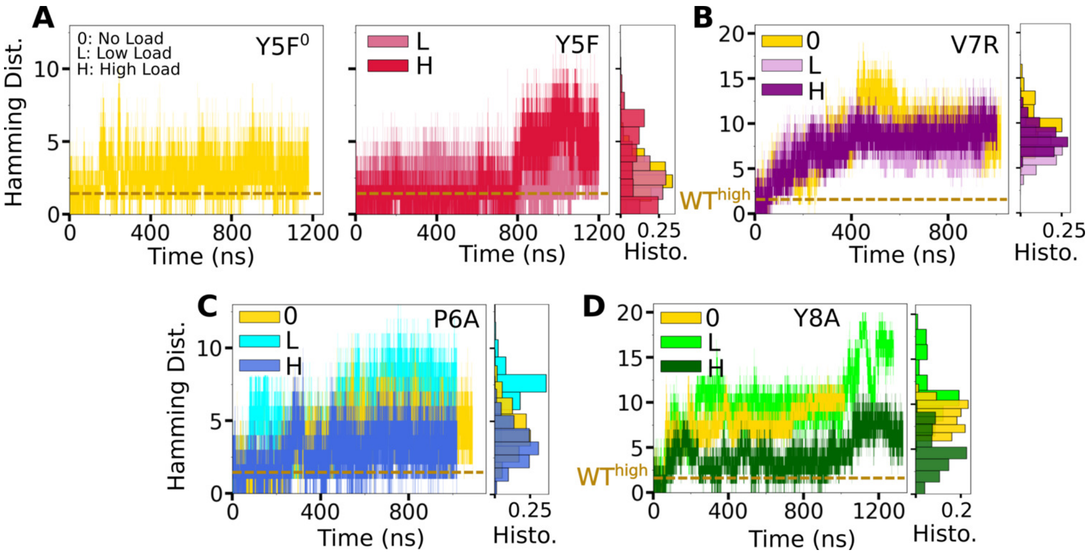

Figure 5—figure supplement 1. Trajectories of $\mathcal{H}$ for mutant complexes. (A,B) Modified agonists. (C,D) Antagonists. The same cutoff criteria were used to calculate initial contacts as in **Figure 2**B. Data after 500 ns were used for histograms on the right of each panel.

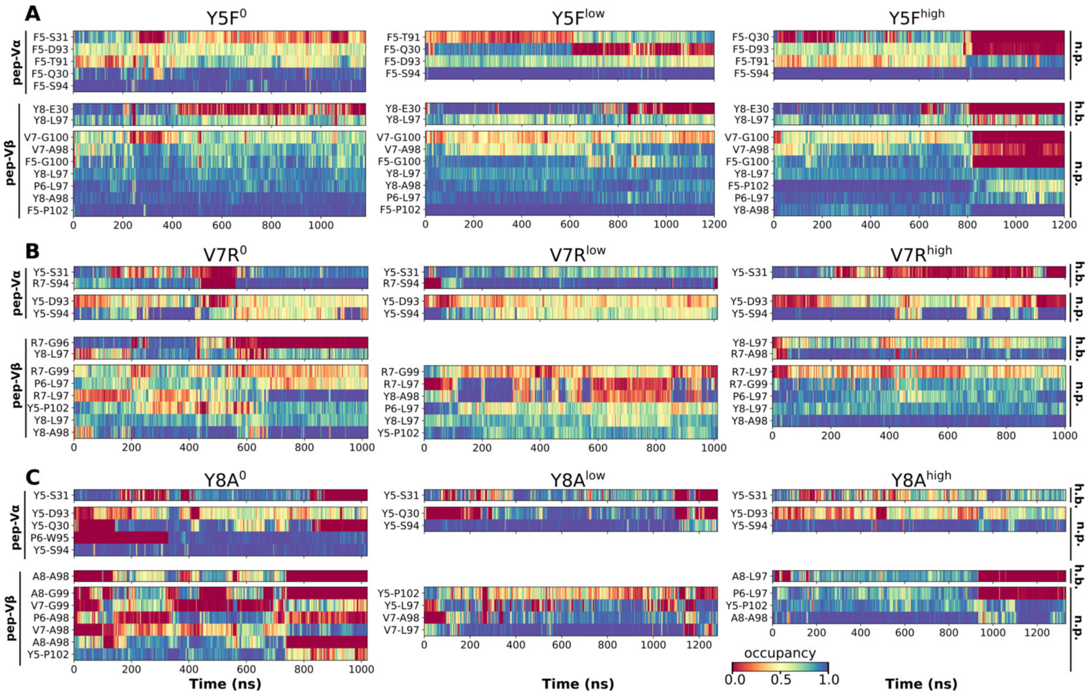

Figure 5—figure supplement 2. Contact occupancy heat maps for residues 5–8 of the mutant peptides. (A) Y5F, (B) V7R, and (C) Y8A. Corresponding heat maps for WT^{high} and P6A are in **Figure 5**C–F.

bioRxiv preprint doi: https://doi.org/10.1101/2023.09.10.557064; this version posted September 13, 2023. The copyright holder for this preprint (which was not certified by peer review) is the author/funder, who has granted bioRxiv a license to display the preprint in perpetuity. It is made available under aCC-BY 4.0 International license.

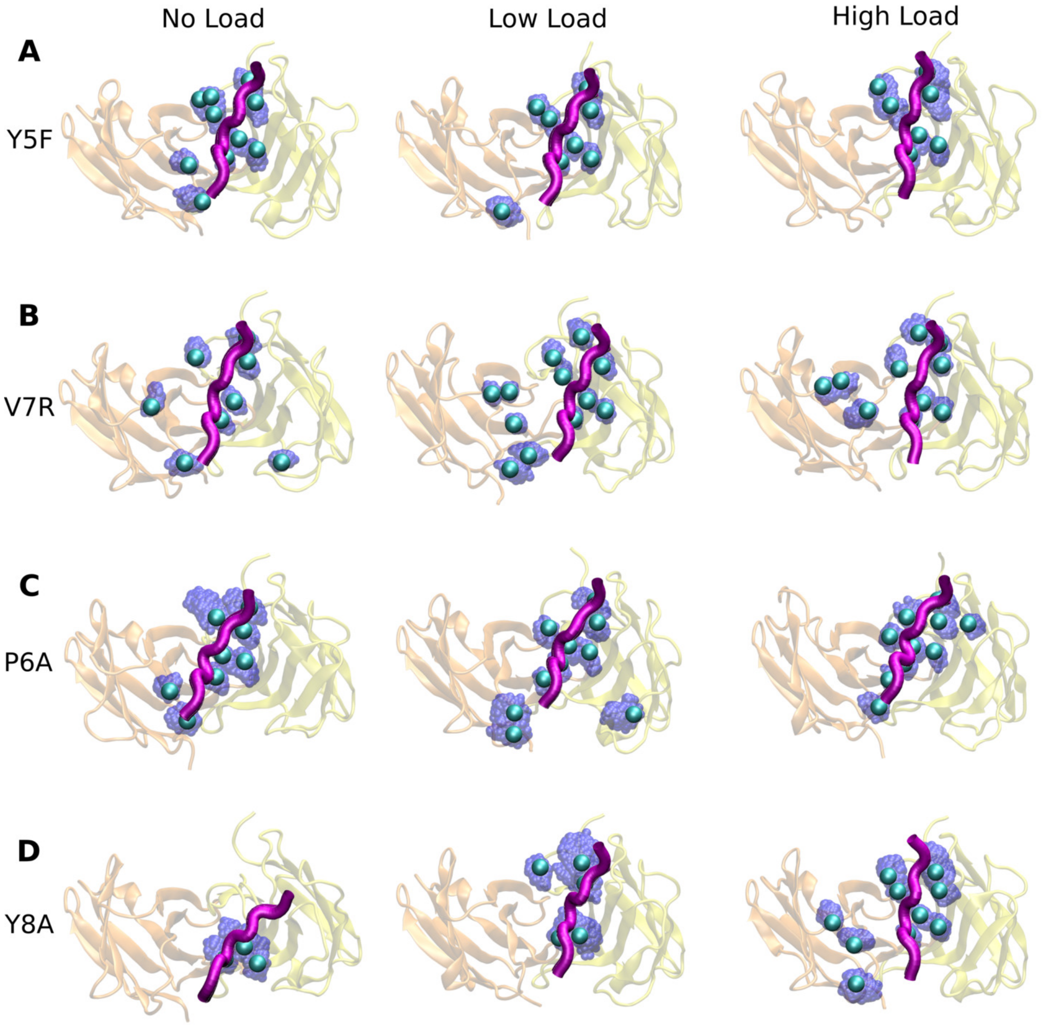

Figure 5—figure supplement 3. Locations of high-occupancy contacts with pMHC in mutant systems. (A) Y5F, (B) V7R, (C) P6A, and (D) Y8A. Compared to $\mathrm{WT}^{\mathrm{high}}$ or V$\alpha\beta$-pMHC (**Figure 2**F), contacts are overall unevenly distributed or dispersed. The same occupancy cutoffs as in **Figure 2**F were used for selecting residues.

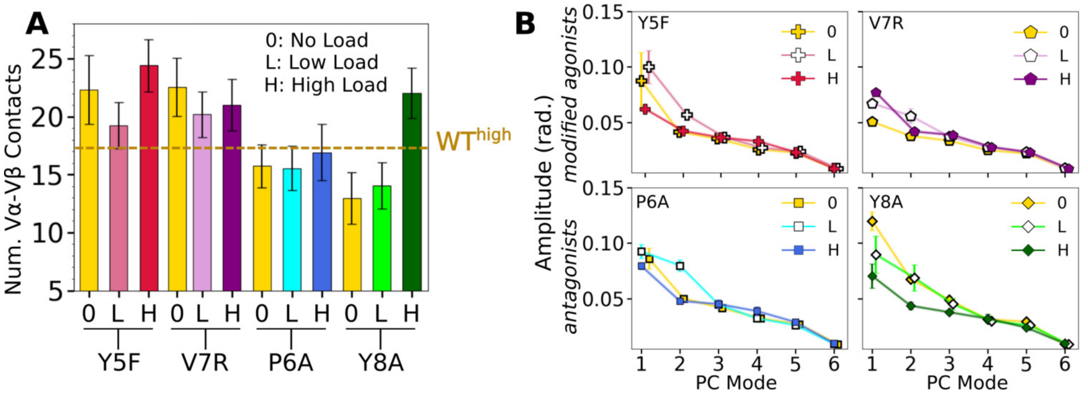

Figure 6—figure supplement 1. Vα-Vβ motion of mutant systems. (A) Number of Vα-Vβ contacts, counted in the same way as in **Figure 3**A. Dashed line is the average for $\mathrm{WT}^{\mathrm{high}}$. (B) V$\alpha$-V$\beta$ PC amplitudes. Calculated the same way as in ***Figure 3—figure Supplement 1***A.

bioRxiv preprint doi: https://doi.org/10.1101/2023.09.10.557064; this version posted September 13, 2023. The copyright holder for this preprint (which was not certified by peer review) is the author/funder, who has granted bioRxiv a license to display the preprint in perpetuity. It is made available under aCC-BY 4.0 International license.

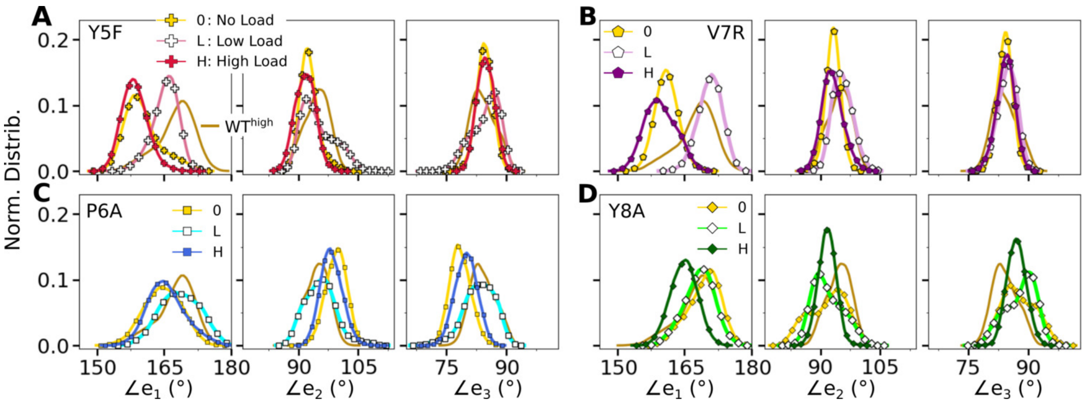

Figure 6—figure supplement 2. Distribution of triad arm angles in mutant systems. (A,B) Modified agonists and (C,D) antagonists. Respective plot for WT^{high} in **Figure 3**C is included in all panels (without markers) for comparison.

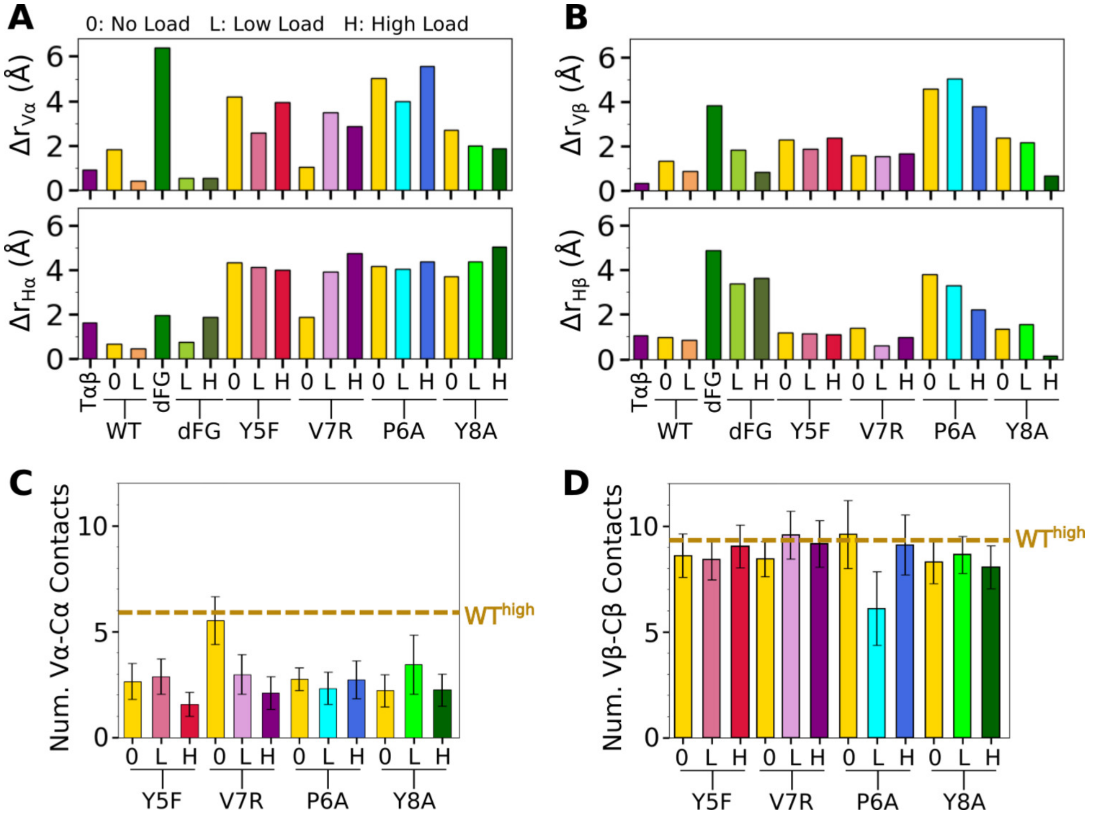

Figure 6—figure supplement 3. Comparison of mutant average V-C BOCs and interfaces with those of WT^{high}. All BOCs are aligned to the C-module of WT^{high}. (A,B) Distances of beads for (A) Vα and Hα, and (B) Vβ and Hβ from those of WT^{high}, revealing the extent of deformation. (C,D) Number of V-C contacts for each chain. Dashed line denotes respective value for WT^{high} in ***Figure 4—figure Supplement 1***B,C.

bioRxiv preprint doi: https://doi.org/10.1101/2023.09.10.557064; this version posted September 13, 2023. The copyright holder for this preprint (which was not certified by peer review) is the author/funder, who has granted bioRxiv a license to display the preprint in perpetuity. It is made available under aCC-BY 4.0 International license.

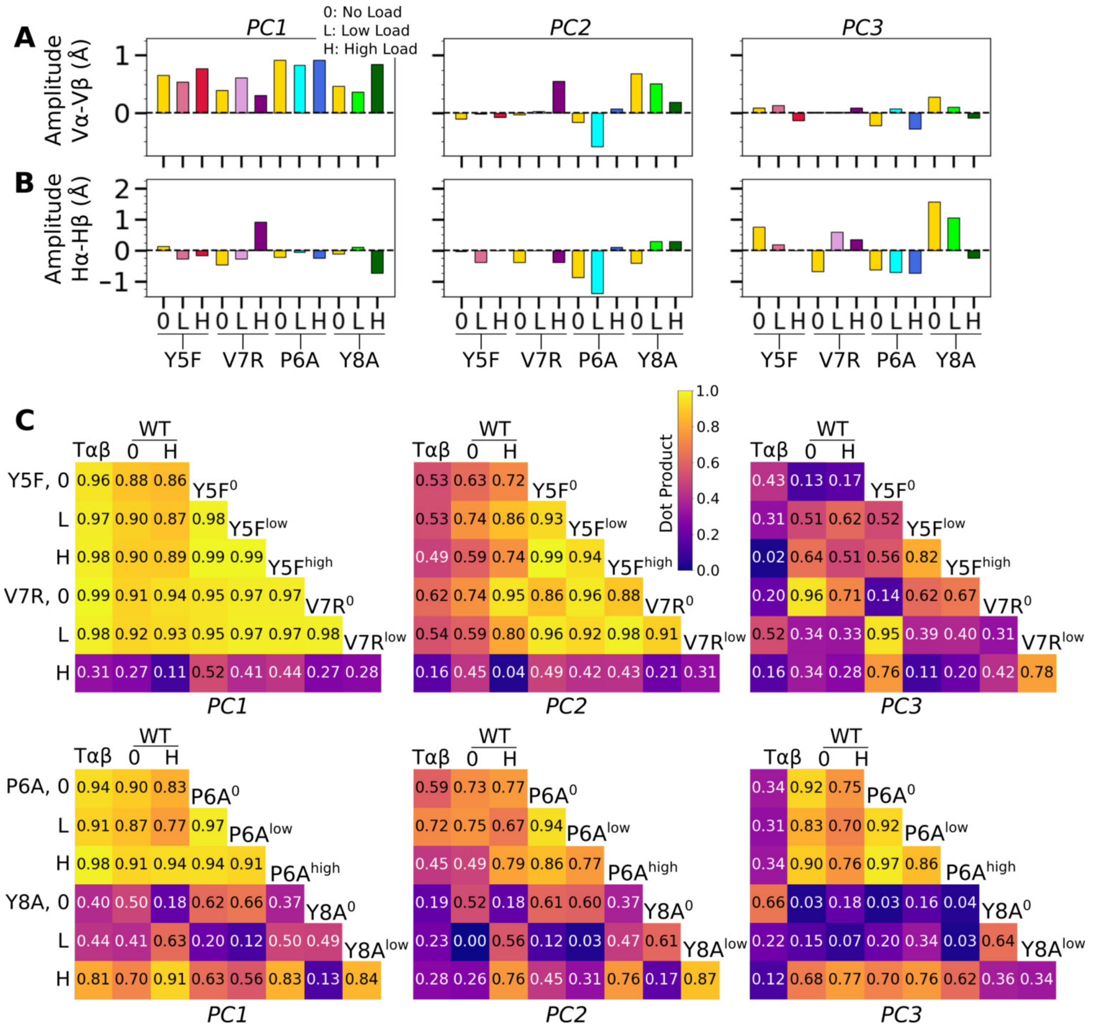

Figure 6—figure supplement 4. V-C motion of mutants. (A,B) Differences in PC amplitude between BOC PC of (A) Vα vs. Vβ and (B) Hα vs. Hβ. Compare with **Figure 4**C for WT systems. (C) Dot products between BOC PC vectors for the listed systems.

bioRxiv preprint doi: https://doi.org/10.1101/2023.09.10.557064; this version posted September 13, 2023. The copyright holder for this preprint (which was not certified by peer review) is the author/funder, who has granted bioRxiv a license to display the preprint in perpetuity. It is made available under aCC-BY 4.0 International license.

885

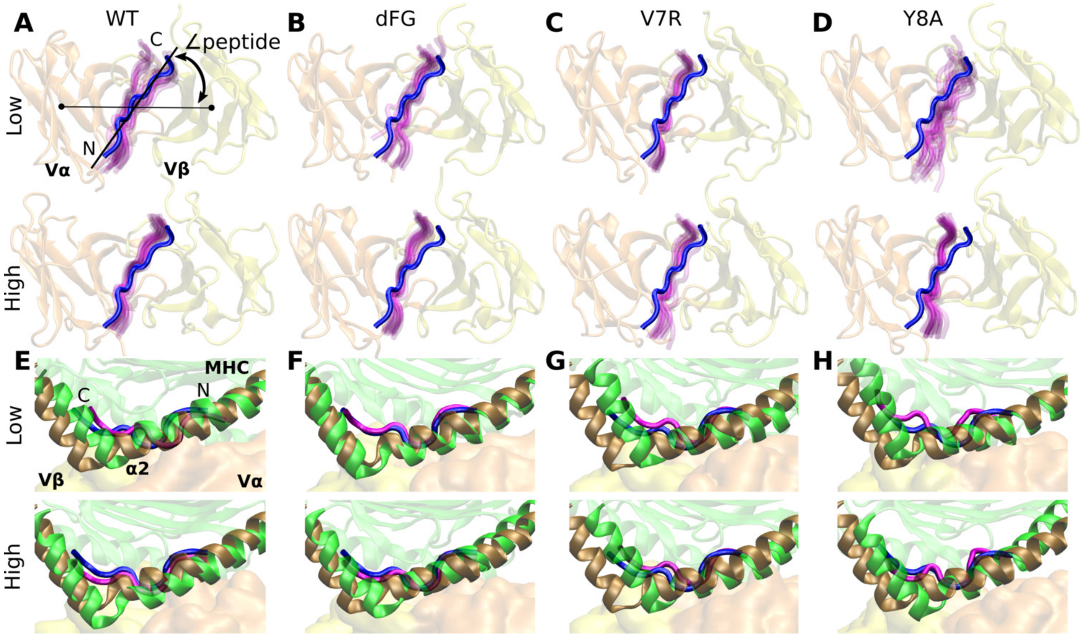

Figure 7—figure supplement 1. Motion at the interface related to ∠peptide. (A–D) View of interface from the top of the V-module. The peptide from the crystal structure (blue) of each respective system is overlaid on frames of the peptide during simulation (magenta) rendered every 50-ns from 500 ns to the end. (E–H) Positional shift of pMHC. Side view showing MHC α2 helix (brown) and peptide (blue) from the crystal structure overlaid with MHC α2 helix (green) and peptide (magenta) of the last rendered frame from panels A–D.
